# Design and construction of multi epitope- peptide vaccine candidate for rabies virus

**DOI:** 10.6026/97320630019167

**Published:** 2023-02-28

**Authors:** Hasanain Abdulhameed Odhar, Ahmed Fadhil Hashim, Suhad Sami Humadi, Salam Waheed Ahjel

**Affiliations:** 1 Department of pharmacy, Al-Zahrawi University College, Karbala, Iraq

**Keywords:** epitope, peptide vaccine, rabies, glycoprotein, docking, dynamics simulation

## Abstract

Rabies virus is a zoonotic pathogen that causes lethal encephalitis with a case fatality rate of almost 100% in unvaccinated individuals. The currently available vaccines against rabies are composed of inactivated viral particles that only confer a short-term immune response. It is well-known that the entry of rabies virus into host cells is mediated by a trimeric glycoprotein presents on the surface of viral envelope. As the sole viral surface protein, this trimeric glycoprotein represents a promising molecular target to design new vaccines and neutralizing antibodies against rabies virus. Epitope mapping studies had identified several antigenic sites on the surface of trimeric pre-fusion glycoprotein of rabies virus. Therefore, it is of interest to screen the rabies virus glycoprotein by different web-based immuno-informatics tools to identify potential B-cells and T-cells linear epitopes. Here, we present a construct of peptide vaccine that consists of these predicted linear epitopes of rabies virus glycoprotein together with appropriate linkers and adjuvant. Various online prediction tools, molecular docking and dynamics simulation assume that the vaccine construct may be stable, safe and effective. However, validation of these in-silico results is necessary both in vitro and in vivo setting.

## Background:

**Figure 1:**
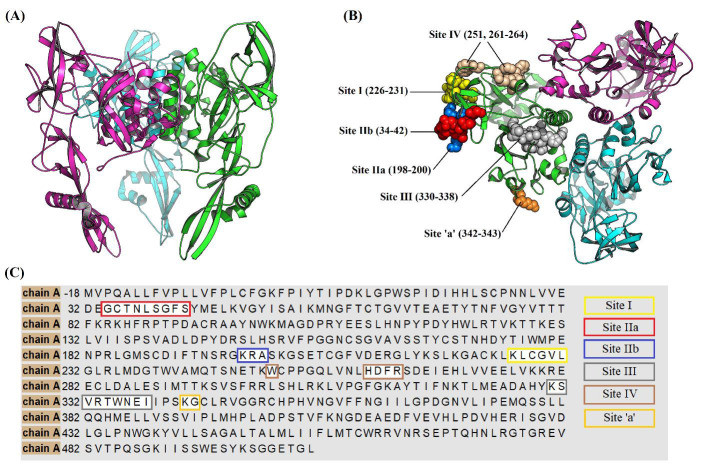
(A) Cartoon illustration for the trimeric pre-fusion rabies virus glycoprotein, PDB: 7U9G. (B) Location and sequence number of the main antigenic sites on the surface of rabies virus glycoprotein trimer. (C) Sequence map for amino acid residues of rabies virus glycoprotein chain A, the locations of antigenic epitopes are highlighted.

Rabies virus is a single-stranded negative-sense RNA virus that belongs to the *Rhabdoviridae* family and the *Lyssavirus* genus [1]. The rabies virus (RABV) is considered a neurotropic virus that causes fatal encephalitis in almost all the mammals, the virus is usually transmitted by the bite of an infected animal. RABV replicates in muscles and travels to the spinal cord and brain through peripheral neurons after bite. When RABV reaches to the brain, it replicates there and spreads to other tissues like salivary glands. Finally, death usually occurs due to dysfunction of neurons [2]. The case fatality rate of rabies disease in the unvaccinated persons is nearly 100%. Every year, RABV results in the death of 50,000 - 70,000 individuals worldwide. In fact, there is no successful treatment against rabies disease when the clinical signs starts appearing [3]. Clinically, human rabies can be manifested by either the hyperactive furious form or the less commonly recognized paralytic form [4]. The incubation period for RABV is comparatively long and it usually ranges between 1 and 3 months, therefore post exposure prophylaxis (PEP) is considered highly effective in averting rabies disease [5]. As such, the unvaccinated individuals should receive PEP once exposed to RABV. The currently available PEP tools against rabies includes: washing and flushing of infected wound, administration of polyclonal antibodies and multiple doses of rabies vaccine [6]. The available rabies vaccines are composed of inactivated RABV particles and these vaccines don't provide a lifelong protection. It has been observed that the level of neutralizing antibodies usually wanes 1 - 5 years after administration of rabies vaccine [7]. Development of more modern rabies vaccines that are based on subunit protein or mRNA are still challenging due to difficulties in the production of stable trimeric RABV glycoprotein (RABV-G) [8]. RABV-G is a trimeric viral fusion protein that presents on the surface of RABV envelope. As the only viral surface protein, RABV-G is considered the main target for neutralizing antibodies. Depending on pH value of the medium, RABV-G can undergo a reversible transition between pre-fusion form at neutral pH and post-fusion form at acidic pH [9,10]. It is believed that the trimeric pre-fusion RABV-G is the perfect target to design new vaccines or neutralizing antibodies as it displays the main known epitopes [11]. Former epitope mapping studies have detected three main antigenic sites on the surface of RABV-G designated as site I to III. In addition, two less common antigenic sites were also recognized and named as site IV and site 'a' [12]. The location and sequence of these five antigenic sites on the surface of RABV-G trimer can be seen in Figure 1. In this trend, several human monoclonal antibodies (mAbs) against RABV-G were isolated and characterized. Some of these antibodies like 17C7 and RVA122 were able to neutralize many isolates of rabies virus through binding to epitopes within the antigenic site III of RABV-G [13, 14]. Recently, the use of 17C7 and RVA122 antibodies has led to the successful crystallization of RABV-G trimer in the pre-fusion state [15,16]. As mentioned before, the isolation of RABV-G in the trimeric pre-fusion form can greatly help efforts to design better vaccines and neutralizing antibodies against rabies virus [8]. In this in-silico study, several immuno-informatics tools were employed to predict and evaluate linear epitopes on the surface of RABV-G crystal. Then, these potential antigenic epitopes were used to design and construct a multiepitope-based peptide vaccine for potential use against RABV.

##  Methodology:

## Setting up immuno-informatics plan:

**Figure 2:**
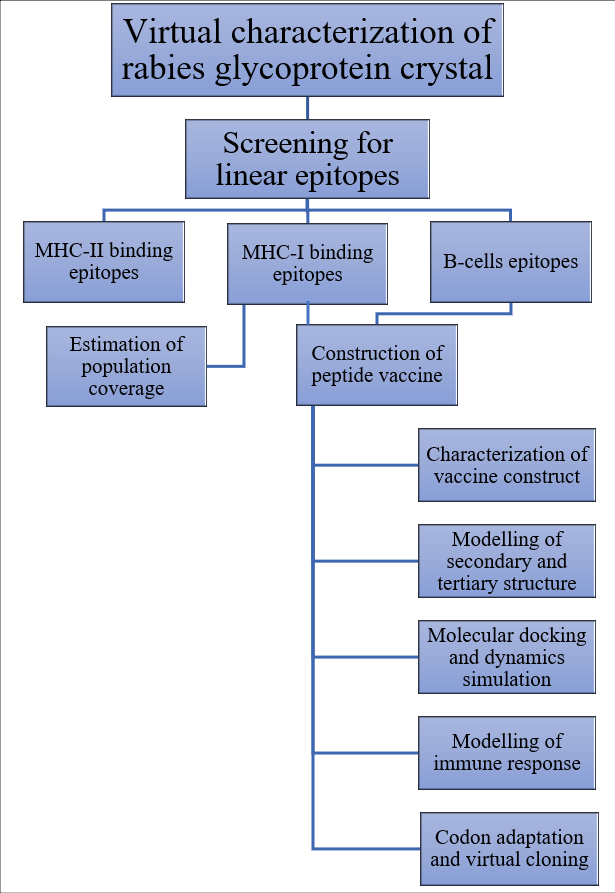
A schematic overview for major steps of the immuno-informatics study.

A schematic representation for main stages of this immuno-informatics study is summarized in Figure 2.

## Prediction of RABV-G immunologic and physicochemical features:

The primary sequence of RABV-G chain A was downloaded as FASTA file from the Protein Data Bank with PDB code: 8A1E [17]. At first, this sequence of amino acids was submitted to the DeepTMHMM web-based tool in order to predict number of transmembrane helices [18]. The sequence of RABV-G chain A was also uploaded to VaxiJen v2.0 [19], AllerTOP v2.0 [20] and VirulentPred [21] online tools to anticipate antigenicity, allergenicity and virulence potentials for target protein. The threshold used for antigenicity prediction by VaxiJen tool was 0.5. And in order to avert any possibility of auto-immune reaction, the protein sequence was assessed for homology with human proteins by using BLASTP online tool [22]. Lastly, the FASTA sequence of RABV-G monomer was submitted to the ProtParam online program to compute several physicochemical properties of this protein [23].

## Linear epitopes prediction and characterization:

Both B-cells and T-cells linear epitopes were predicted based on RABV-G chain A residues sequence by using the Immune Epitope Database (IEDB) website [24]. For the prediction of linear B-cells epitopes, the sequence of residues in chain A of RABV-G was submitted to the antibody epitope prediction tool in IEDB website. Here, the Bepipred Linear Epitope Prediction 2.0 method was employed to predict B-cells epitopes and default threshold of 0.5 was used for interpretation of final results. It is worth to mention that the Bepipred Linear Epitope Prediction 2.0 method depends on an algorithm that was trained on epitopes of known antigen-antibody complexes [25]. Then, the sequence of RABV-G chain A was uploaded as FASTA file to the MHC-I binding prediction tool in IEDB server in order to anticipate linear T-cells epitopes presented by major histocompatibility complex class I (MHC-I). For this purpose, the NetMHCpan EL 4.1 method was applied to calculate the binding potential of antigenic peptides to MHC-I molecules. The algorithm of NetMHCpan was trained on a large set of quantitative information about MHC-I binding [26]. By using a reference set of 54 Human Leukocyte Antigen (HLA) alleles available in IEDB site, the MHC-I binding peptides were sorted according to their percentile rank. After that, the linear T-cells epitopes presented by the major histocompatibility complex class II (MHC-II) were anticipated by using MHC-II binding prediction tool available through IEDB server. To anticipate MHC-II binding peptides in RABV-G chain A, the IEDB recommended 2.22 prediction method was employed where the prediction tool attempts to use the best method for a given MHC molecule like SMM-align [27], Sturniolo [28] and NetMHCIIpan [29] methods. A panel of 27 HLA alleles available in IEDB site was used to predict the MHC-II binding peptides with a default length of 15 mer. Again, these MHC-II binding peptides were arranged according to their percentile rank. Finally, both B-cells and T-cells linear epitopes were characterized by prediction of their antigenicity [19], allergenicity [20], water solubility [30], toxicity [31] and interferon-gamma (INF-γ) induction capacity [32]. These B-cells and T-cells epitopes were filtered and selected based on their predicted characteristics.

## Estimation of population coverage for the selected T-cells epitopes:

The major histocompatibility complex (MHC) molecules are greatly polymorphic and it is expressed at various frequencies throughout different populations. In order to increase the population coverage, the peptide vaccine should have several epitopes with diverse HLA binding specificities. For this purpose, the population coverage tool available in IEDB website to anticipate the response of different populations in the world to the selected two T-cells epitopes [33]. We have assigned the number of epitopes to 2 peptides, then different areas in the world were chosen for the assessment of population coverage. Class I separate option was used to account for MHC class I restricted T- cells epitopes. For MHC class I peptides, we have used the following alleles: A*01:01, A*02:01, A*02:06, A*03:01, B*07:02, A*11:01, A*23:01, A*26:01, B*35:01, B*51:01.

## Construction of peptide vaccine:

The design of the peptide vaccine did involve linking the selected B-cells and T-cells epitopes by using the AAY linker. This type of flexible linker can reduce the formation of junctional epitopes and increase the formation of natural epitopes [34]. Then, Cholera toxin B subunit was added with EAAAK linker to the N-terminus of peptide vaccine in order to enhance immunogenicity of the construct. The rigid EAAAK linker can provide adequate separation between epitopes and the adjuvant of Cholera toxin B subunit [35].

## Prediction of peptide vaccine immunologic and physicochemical features:

The amino acids sequence for peptide vaccine was submitted to the ProtParam online tool to predict different physicochemical properties of the construct [23]. The antigenicity, allergenicity and virulence potentials for the vaccine construct were assessed by different web-based tools [19, 21].

## Modeling of the secondary and tertiary structure for peptide vaccine:

Then, the peptide vaccine residues sequence was submitted to the PSIPRED online tool to anticipate the secondary structure of the construct [36]. Also, the tertiary structure of the vaccine was predicted by using the 3Dpro tool in SCRATCH protein predictor website [37]. The generated tertiary structure was further improved by employing the Galaxy refine server [38]. Finally, the refined tertiary structure for vaccine construct was validated by Ramachandran plot [39]. This plot can provide a view about protein conformation by assessing the torsional angle for each residue [40].

## Molecular docking and dynamics simulation analysis:

The refined tertiary structure of vaccine construct was docked into Toll-like receptor 8 (TLR8) protein by using HDOCK server [41]. This web-based server utilizes a unique strategy in which experimental data about protein–protein binding site is included during docking process [42]. The TLR plays an essential role in connecting innate immunity with adaptive immunity, and TLR8 is specifically important in detecting single stranded RNA viruses [43,44]. For this docking study, only chain A of TLR8 crystal with PDB code of 5Z14 was submitted to HDOCK server [45]. The output of HDOCK server lists the best ten docking complexes, and complex 1 has the highest docking score (more negative) with greatest confidence score. This top complex was then downloaded for further evaluation by PyMOL version 2.3 [46] and molecular dynamics (MD) simulation. For the MD study, the best docking complex between vaccine and TLR8 chain A was subjected to simulation for 20 nanoseconds by using YASARA Dynamics v20.12.24 [47]. Here, the applied MD simulation procedure is similar to what we used in our previously published articles [48, 49, 50]. The simulation output was then analyzed by evaluating the root mean square fluctuation (RMSF) for TLR8-peptide vaccine complex during simulation period.

## Virtual simulation of immune response to peptide vaccine constructs:

The capacity of the proposed multiepitope-based peptide vaccine to activate different components of host immune system was modelled by using C-ImmSim server [51]. For this step, the vaccine construct sequence was submitted in FASTA format. The simulation steps were specified to 1000 so that immune response can be modelled for a duration of 333 days. During this simulation of immune response, two injections of peptide vaccine were applied at time steps of 1 and 180 which are equivalent to 1 and 60 days in real life respectively. Other parameters of simulation were set to default values.

## Codon adaptation and virtual cloning:

The amino acids sequence for peptide vaccine was reversibly translated into the corresponding DNA sequence with the application of codon adaptation tool (JCAT) [52]. The adaptation of codon can ensure efficient expression in organisms like Escherichia coli strain K12. Then, the optimized DNA sequence of vaccine construct was cloned computationally into pET-53-DEST expression vector by using SnapGene tool.

## Results and Discussion:

**Figure 3:**
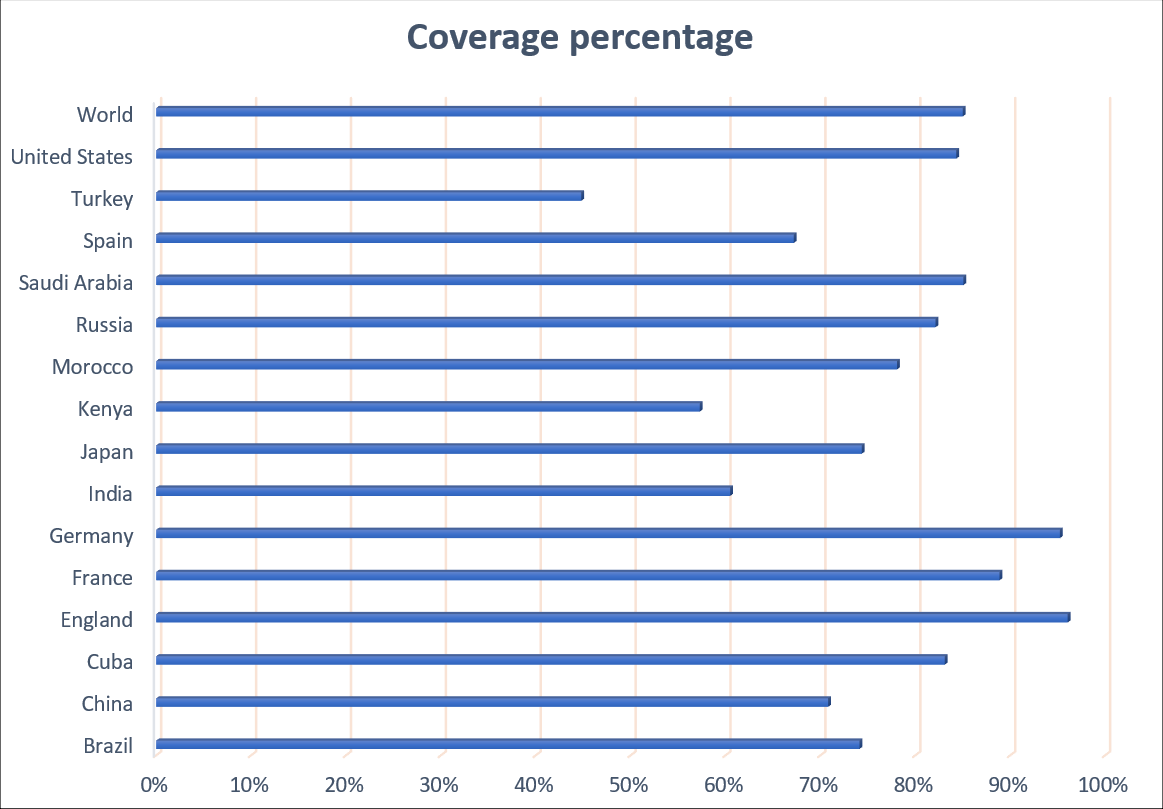
Percentage of population coverage for the selected T-cells epitopes based on MHC-I restricted alleles.

**Figure 4:**
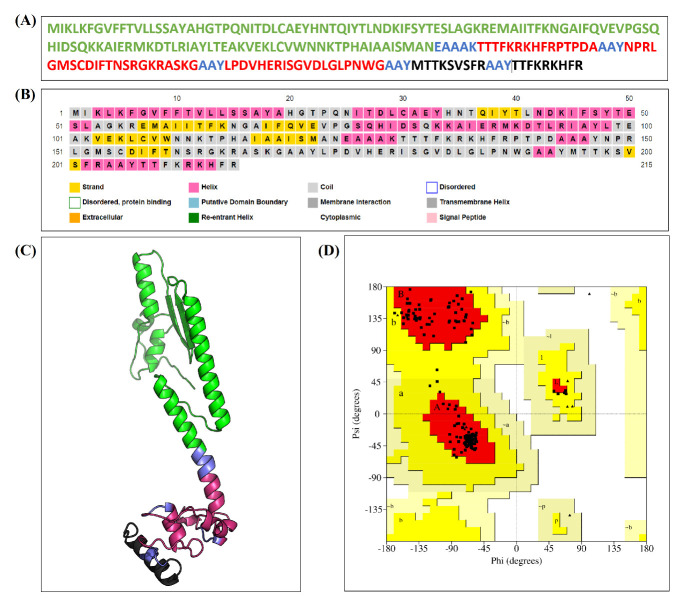
An overview for the multiepitope-based peptide vaccine construct. (A) The amino acids sequence of vaccine construct in one letter format. (B) An illustration for the secondary structure analysis of peptide vaccine. (C) A graphical representation for the refined tertiary structure of peptide vaccine. (D) Ramachandran plot analysis of the refined tertiary structure of peptide vaccine. In both (A) and (C), the Cholera toxin B subunit is shown by green color while B-cells and T-cells epitopes are illustrated as red and black colors respectively. Both AAY and EAAAK linkers are represented by blue color.

**Figure 5:**
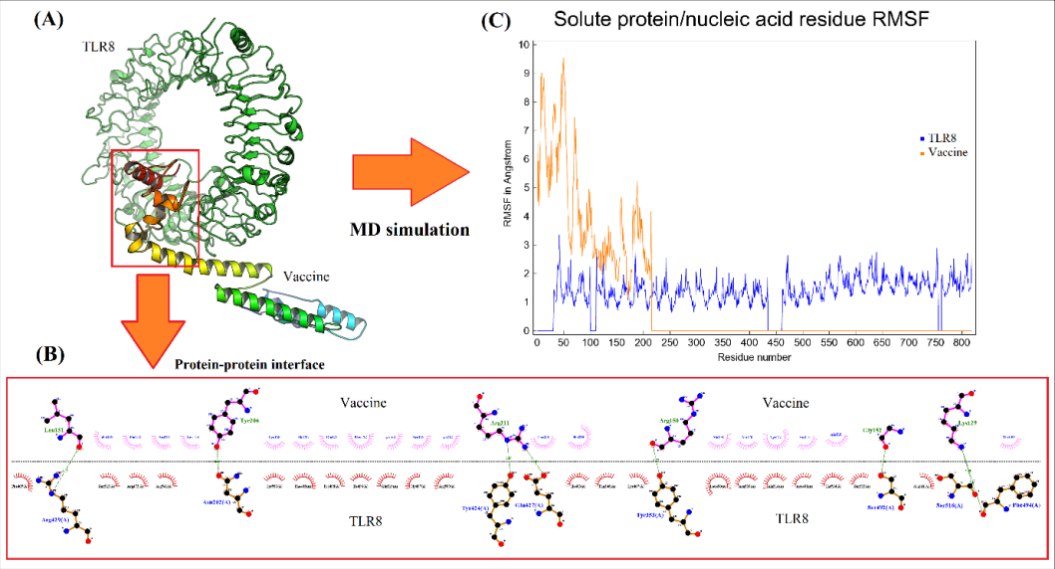
Molecular docking and dynamics simulation of vaccine construct against TLR8. (A) Three-dimensional image for the docking of best complex between peptide vaccine and TLR8. (B) Two-dimensional overview for the interaction interface between peptide vaccine and TLR8 in the best docking complex. (C) The root mean square fluctuation (RMSF) for every residue of TLR8 and vaccine construct during simulation.

**Figure 6:**
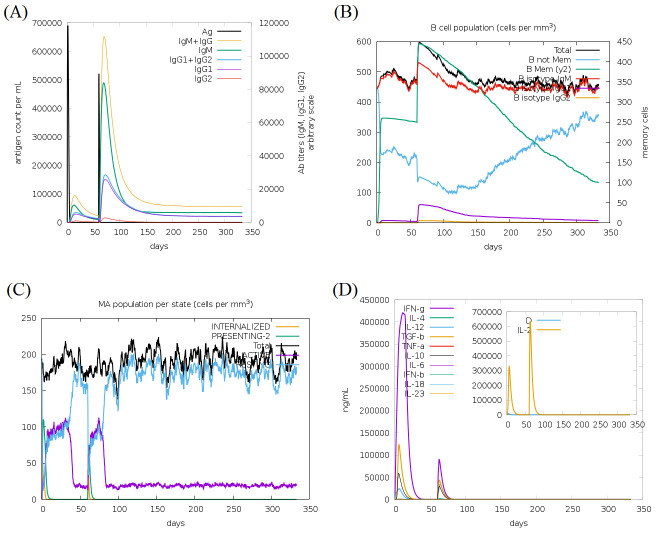
Modelling of host immune response after administration of two doses of peptide vaccine. (A) Immunoglobulins and antigen levels, (B) B-cells population level, (C) Macrophages level per activity state and (D) Level of cytokines and interleukins.

**Figure 7:**
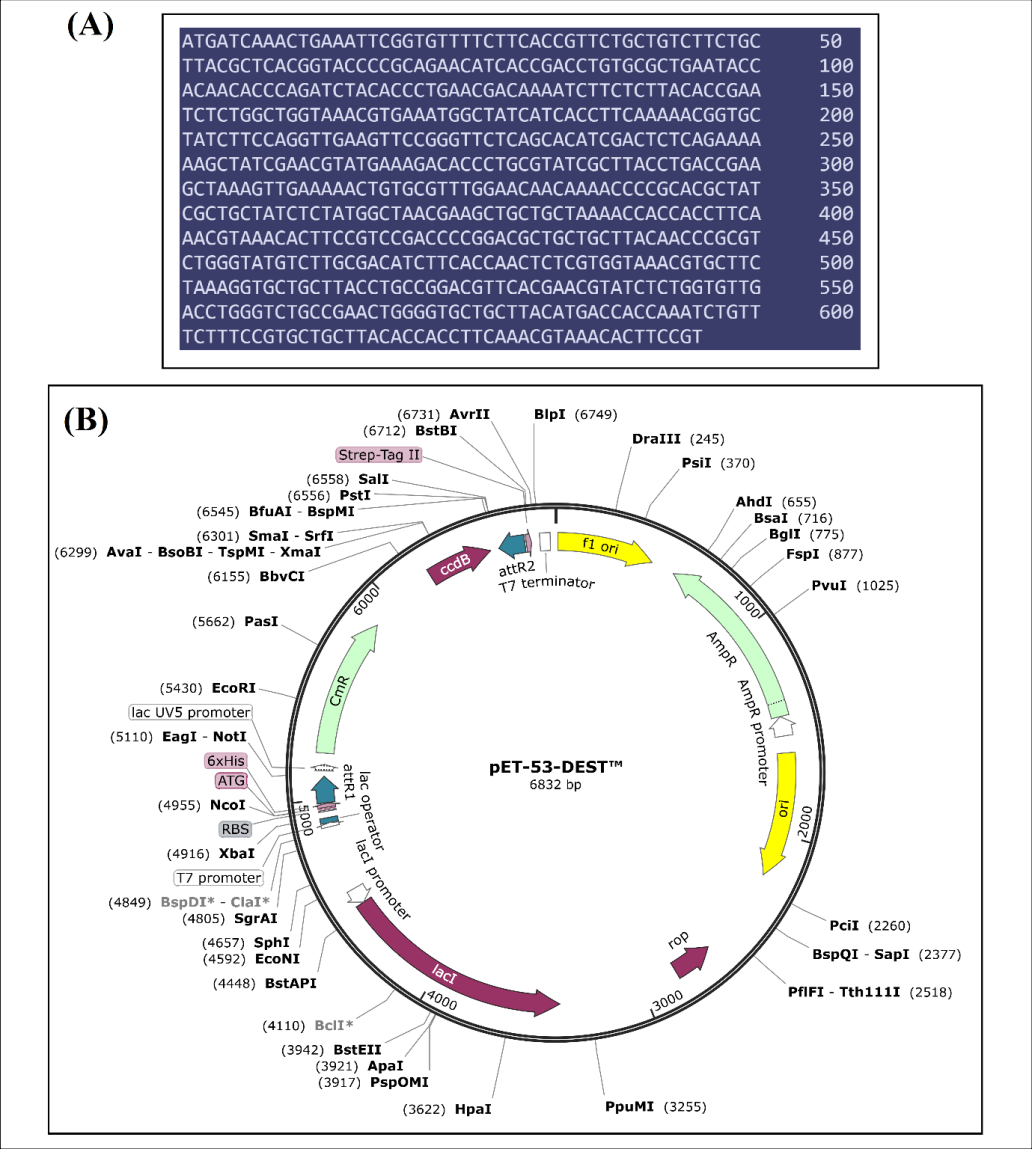
Codon adaptation and virtual cloning of vaccine construct. (A) Optimized DNA sequence of peptide vaccine and (B) Cloning of vaccine DNA sequence into expression vector.

In order to design and construct an effective vaccine candidate, the employed target protein must be localized on the surface of pathogen in a solvent accessible region that facilitate the identification and recognition of potential epitopes by B-cells membrane bound immunoglobulins [53,54]. Also, the target protein should have no more than one trans-membrane helix and without significant sequence homology with human proteins. Additionally, the target protein of designated pathogen should be non-allergenic with antigenicity and virulence potentials greater than 0.5. Finally, the molecular weight of the used protein should be less than 110 kDa [55]. And by evaluating the predicted immunogenic and physicochemical properties of RABV-G chain A in Table 1, we can note that this protein has only one transmembrane helix. Also, the protein has antigenicity and virulence potentials greater than 0.5 and it is probably non-allergenic. Additionally, the possibility of auto-immune reaction for RABV-G chain A is probably low due to no significant similarity with human proteins. And through assessment of physicochemical properties for RABV-G chain A in Table 1, we can see that the molecular weight of the protein is less than 110 kDa. According to Table 1, the monomer A of RABV-G is probably a neutral protein with isoelectric point (PI) of 7.33 and the number of positively charged residues is equal to the negatively charged ones. It is known that the isoelectric point (PI) is defined as the solution pH in which the target protein has a neutral electric charge [56]. Unfortunately, the RABV-G chain A may be unstable protein as the predicted instability index is greater than 40 [57]. Therefore, the use of the whole RABV-G may be challenging in designing new vaccine candidates due to possible protein instability. As such detection of potential linear epitopes on the surface of RABV-G monomer maybe a more convenient approach to design a stable and effective multiepitope-based peptide vaccine candidate. The list of linear B-cells and T-cells epitopes predicted on the surface of RABV-G chain A can be seen in Table 2. As noted in this table, the predicted epitopes were filtered and shortlisted through evaluation of several characteristic scores regarding antigenicity, allergenicity, water solubility, toxicity and ability to induce the release of INF-γ. According to these descriptive characteristics in Table 2, only three B-cells epitopes and two MHC-I binding epitopes were selected for the construction of peptide vaccine candidate. The selected B-cells epitopes include peptides number 3, 6 and 16 while the MHC-I binding epitopes are peptides number 9 and 11. It is worth to mention that the B-cells epitope number 3 shares a sequence similarity with the MHC-I binding epitope number 11 as noted in Table 2. The yellow color seen in Table 2 indicates unfavorable character for each epitope. Then, the two T-cells epitopes presented by MHC-I pathway were assessed by the population coverage online tool. As presented in Figure 3, these two epitopes show good population coverage with worldwide coverage percentage of 85%. As such, these two T-cells epitopes (9 and 11) are appropriate for the construction of a peptide vaccine candidate and can reduce MHC-I restriction of T-cells responses. An overview for the construct of peptide vaccine can be seen in Figure 4, where the vaccine design did involve linking the epitopes together by using AAY linkers. The Cholera toxin B subunit was fused to the N-terminus of vaccine as adjuvant to enhance antigenicity of the peptide. The rigid EAAAK linker was employed to add Cholera toxin B subunit to the peptide vaccine. The amino acids sequence of this multiepitope-based peptide vaccine candidate is illustrated in Figure 4 (A). Analysis of the secondary structure for the peptide vaccine as seen in Figure 4 (B) indicated that the percentage of β-strand, α-helix and coil are 17, 36 and 47 respectively. Also, the refined tertiary structure of vaccine construct can be seen in Figure 4 (C). This refined structure had successfully passed the Ramachandran plot analysis as noted in Figure 4 (D) in which 94.8% of the residues are in the most favored regions and only 5.2% of the residues are in the additional allowed regions of the model. After that the peptide vaccine sequence was further validated to predict the construct’s physicochemical properties, antigenicity, allergenicity and virulence capacity as summarized in Table 3. Based on this table, the vaccine construct is predicted to be immunogenic with antigenicity and virulence potential greater than 0.5. Also, the vaccine construct is probably non-allergenic. The net charge of this peptide vaccine is positive as the number of positively charged residues is greater than the negatively charged ones. Also, the isoelectric point (PI) is more than 7 which indicates that the construct charge is positive. Finally, the design of peptide vaccine may be stable as the instability index in Table 3 is less than 40. According to molecular docking study, the construct of peptide vaccine is very likely to bind to TLR8 protein because the docking score for the top complex was -401.18 which is better than the cutoff limit of -200 as specified by HDOCK server. In addition, the confidence score for the top complex was 0.99 which is higher than the limit of 0.7 as noted in HDOCK server guideline. Analysis of TLR8-vaccine complex interface for the top docking model indicates that about 16% of vaccine residues are involved in interaction with 5.97% of TLR8 residues at a distance less than 5 Angstrom. An overview for molecular docking and dynamics simulation of peptide vaccine against TLR8 can be seen in Figure 5. Based on the assessment of RMSF value for each residue of vaccine-TLR8 complex in Figure 5 (C), it is evident that the vaccine construct exhibits a higher flexibility than TLR8. The residues of vaccine construct show a higher fluctuation in position from its reference position (simulation time averaged position). The modelling of host immune response to the proposed peptide vaccine construct can be observed in Figure 6. As seen in this figure, the administration of two doses of peptide vaccine with duration of 2 months between these two doses can generate a long-lasting immune response. According to Figure 6 (A), the level of immunoglobulins is significantly elevated after the vaccine second dose administration. Also, the level of memory B-cells is significantly increased after vaccine administration, but it declines afterward as noted in Figure 6 (B). While the level of B-cells that produce IgG1 is remarkably high after the second dose only. In addition, there is an increase in activity and antigen presentation of macrophage after vaccine administration as noted in Figure 6 (C). Finally, the level of INF-γ is greatly elevated especially after the first dose of vaccine While the level of interleukin-2 (IL-2) rises mostly after the second dose as shown in Figure 6 (D). In the last stage of this in-silico project, the amino acids sequence of peptide vaccine was backward translated into DNA sequence with adaptive optimization of the codon to ensure efficient expression in Escherichia coli microorganism. The optimized DNA sequence can be seen in Figure 7 (A) and it consists of 645 nucleotides. This nucleotides sequence was then computationally cloned into an expression vector as presented in Figure 7 (B).

## Conclusion:

Here, we report a peptide vaccine construct with multiple linear B-cells and T-cells epitopes for rabies virus glycoprotein. The construct appears to be stable, safe and effective according to different prediction tools, molecular docking and dynamics simulation. However, further validation in vivo and in vitro is required to confirm these in-silico findings.

## Figures and Tables

**Table 1:** Immunologic and physicochemical characteristics predicted for chain A of rabies virus glycoprotein.

**Property**	**Computed value**
Number of transmembrane helices	1
Antigenicity	0.514
Virulence factor	0.976
Allergenicity	Probable non-allergen
Sequence homology with human	No significant similarity
Number of residues	505
Molecular weight	56.422 kDa
Isoelectric point (PI)	7.33
Negatively charged residues	54
Positively charged residues	54
Instability index	42.34

**Table 2:** A tabular description of B-cells and T-cells linear epitopes predicted on the surface of rabies virus glycoprotein chain A

**No.**	**Start**	**End**	**Peptide sequence**	**Length Antigenicity**		**score Allergenicity scor**	**e Water solub**	**lity INF-γ respons**	**e Toxicity**
**B-cel 1 s linear epitopes**
	6	20	TIPDKLGPWSPIDIH	15	1.33	Non-allergen	Good	Negative	-
2	22	57	LSCPNNLVVEDEGCTNLS	36	0.62	Non-allergen	Poor	-	-
			GFSYMELKVGYISAIKMN						
3	79	93	TTTFKRKHFRPTPDA	15	1.18	Non-allergen	Good	Positive	Negative
4	98	127	YNWKMAGDPRYEESL	30	0.64	Non-allergen	Good	N/A	-
			HNPYPDYHWLRTVKT						
5	140	172	ADLDPYDRSLHSPVFPG	33	0.45	-	-	-	-
			GNCSGVAVSSTYCSTN						
6	182	203	NPRLGMSCDIF	22	0.77	Non-allergen	Good	Positive	Negative
			TNSRGKRASKG						
7	214	220	GLYKSLK	7	-0.51	-	-	-	-
8	248	253	ETKWCP	6	2.22	Non-allergen	Good	Negative	-
9	262	267	DFRSDE	6	0.79	Allergen	-	-	-
10	294	299	KSVSFR	6	0.03	-	-	-	-
11	301	313	LSHLRKLVPGFGK	13	0.02	-	-	-	-
12	334	357	TWNEIIPSKGCLR	24	0.68	Allergen	-	-	-
			VGGRCHPHVNG						
13	370	376	NVLIPEM	7	-0.32	-	-	-	-
14	386	401	ELLVSSVIPLMHPLAD	16	0.08	-	-	-	-
15	406	418	FKNGDEAEDFVEV	13	0.63	Allergen	-	-	-
16	420	438	LPDVHERISGVDLGLPNWG	19	0.95	Non-allergen	Good	Positive	Negative
17	463	502	RVNRSEPTQHNLRGTGREV	40	0.36	-	-	-	-
			SVTPQSGKIISSWESHKSGGE						
**T-cells linear epitopes presented by MHC-I pathway.**
1	486	494	QSGKIISSW	9	-0.25	-	-	-	-
2	5	14	YTIPDKLGPW	10	0.91	Non-allergen	Poor	-	-
3	69	77	ETYTNFVGY	9	0.02	-	-	-	-
4	412	420	AEDFVEVHL	9	0.59	Allergen	-	-	-
5	292	300	TTKSVSFRR	9	0.82	Non-allergen	Good	Negative	-
6	170	178	STNHDYTIW	9	0.49	-	-	-	-
7	397	406	HPLADPSTVF	10	-0.19	-	-	-	-
8	417	426	EVHLPDVHER	10	1.02	Non-allergen	Good	Negative	-
9	291	299	MTTKSVSFR	9	1.19	Non-allergen	Good	Positive	Negative
10	179	187	MPENPRLGM	9	0.92	Non-allergen	Good	Negative	-
11	80	88	TTFKRKHFR	9	0.94	Non-allergen	Good	Positive	Negative
12	333	342	RTWNEIIPSK	10	0.05	-	-	-	-
13	352	360	HPHVNGVFF	9	0.47	-	-	-	-
14	66	74	TEAETYTNF	9	0.34	-	-	-	-
15	434	442	LPNWGKYVL	9	-0.12	-	-	-	-
16	205	213	ETCGFVDER	9	1.05	Allergen	-	-	-
17	306	315	KLVPGFGKAY	10	-0.24	-	-	-	-
18	234	242	RLMDGTWVA	9	0.5	Allergen	-	-	-
19	138	147	SVADLDPYDR	10	1.49	Allergen	-	-	-
20	90 98		TPDACRAAY	9	-0.64	-	-	-	-
**T-cells linear epitopes presented by MHC-II pathway.**
1	438	452	GKYVLLSAGALTALM	15	0.48	-	-	-	-
2	213	227	RGLYKSLKGACKLKL	15	0.3	-	-	-	-
3	294	308	KSVSFRRLSHLRKLV	15	0.18	-	-	-	-
4	376	390	MQSSLLQQHMELLVS	15	0.31	-	-	-	-
5	259	273	NLHDFRSDEIEPLVV	15	0.7	Allergen	-	-	-
6	237	251	DGTWVAMQTSNETKW	15	0.68	Allergen	-	-	-
7	309	323	PGFGKAYTIFNKTLM	15	-0.09	-	-	-	-
8	45	59	ELKVGYISAIKMNGF	15	0.84	Allergen	-	-	-
9	74	88	FVGYVTTTFKRKHFR	15	1.04	Non-allergen	Good	N/A	-
10	35	49	CTNLSGFSYMELKVG	15	1.07	Non-allergen	Poor	-	-
**INF-γ:Interferon-gamma; N/A: Not available; MHC: Major histocompatibility complex.**

**Table 3:** Immunologic and physicochemical characteristics predicted for vaccine construct.

**Property**	**Computed value**
Antigenicity	0.649
Virulence factor	1.001
Allergenicity	Probable non-allergen
Number of residues	215
Molecular weight	24.108 kDa
Isoelectric point (PI)	9.77
Negatively charged residues	17
Positively charged residues	31
Instability index	30.94
